# Supplementary material for: Realizing Broadband NIR Photodetection and Ultrahigh Responsivity with Ternary Blend Organic Photodetector
Source: Nanomaterials (Basel). 2022 Apr 18;12(8):1378. doi: 10.3390/nano12081378 (PMC9027253; doi:10.3390/nano12081378)
Supplement: Supplementary file 1 [file nanomaterials-12-01378-s001.zip › nanomaterials-1678697-supplementary.pdf]

nanomaterials

Supporting information

# Realizing Broadband NIR Photodetection and Ultrahigh Responsivity with Ternary Blend Organic Photodetector

**Yang-Yen Yu [1,*] Yan-Cheng Peng [1] Yu-Cheng Chiu [2] Song-Jhe Liu [3] and Chih-Ping Chen [1,*]**

[1] Department of Materials Engineering, Ming Chi University of Technology, New Taipei City 243, Taiwan
[2] Department of Chemical Engineering, National Taiwan University of Science and Technology, Taipei City, Taiwan
[3] Taiwan Thompson Painting Equipment Co., Ltd., New Taipei City 25169, Taiwan
* Correspondence: yyyu@mail.mcut.edu.tw (YYY), cpchen@mail.mcut.edu.tw (CPC)

**Citation:** Yu, Y.-Y.; Peng, Y.-C.; Chiu, Y.-C.; Liu, S.-J.; Chen, C.-P. Realizing Broadband NIR Photodetection and Ultrahigh Responsivity with Ternary Blend Organic Photodetector. *Nanomaterials* **2022**, *12*, 1378. https://doi.org/10.3390/nano12081378

Academic Editor: Maurizio Passacantando

Received: 28 March 2022
Accepted: 13 April 2022
Published: 18 April 2022

**Publisher's Note:** MDPI stays neutral with regard to jurisdictional claims in published maps and institutional affiliations.

**Copyright:** © 2022 by the authors. Licensee MDPI, Basel, Switzerland. This article is an open access article distributed under the terms and conditions of the Creative Commons Attribution (CC BY) license (https://creativecommons.org/licenses/by/4.0/).

## S.1 Device Fabrication

Glass/ITO (resistance: 6.4 Ω $sq^{-1}$) was used as the substrate; the ITO was ultrasonicated with DI water, acetone, and isopropanol in an ultrasonic oscillator (DELTA, DC200H) for 15 min, and then the glass/ITO substrate was cleaned in a Pulp washer (Harrick Plasma, PDC-32G) for 8 min. ZnO (as the hole transport layer) was spin-coated on the ITO (3000 rpm, 30 s) and then annealed (175 °C, 20 min). The active layer (PM6:BTP-eC9 or PM6:BTP-eC9:$PC_{71}BM$) was then spin-coated (1000 rpm, 30 s) onto the surface of ZnO in a glove box ($H_2O$ < 0.5 ppm; $O_2$ < 0.5 ppm). Finally, $MoO_3$ and Ag layers were deposited on the active layer through physical vapor deposition (vapor deposition rate: 0.5–1 Å $s^{-1}$). The structure of the prepared organic photodetector was ITO/ZnO/PM6:C9: $PC_{71}BM$ (D:A + $x$)/$MoO_3$/Ag.

## S.2. Characterization

The current density and voltage of the sensor were measured using a programmable power controller (Keithley model 2636A) in a dark environment. EQE measurements were performed using a solar cell response measurement system (QE-R3011, Enlitech). The LDR was determined using a Thorlab 530-nm light source (M530L4) and a light source driver (DC2200). The measurements were made at the light intensity of 1 mW, with the light intensity through the attenuator gradually being decreased to 10 pW. The standard film was calibrated using Newport UV-818. Water contact angles were investigated using a PSC-100B instrument (Pentad Scientific) to determine the hydrophilicity, hydrophobicity, and surface energy. GIWAXS spectra were recorded using the TLS13A beam of the National Synchrotron Radiation Research Center (NSRRC) in Taiwan. AFM was performed using a Bruker Dimension Edge microscope operated in tapping mode at room temperature. AFM-FFT images were analyzed using Gwyddion software. The response frequency was converted from a photocurrent to a photovoltage by using a low-noise current preamplifier with A $V^{-1}$ gain factor of $10^5$ and no bandwidth filter (Ametek, model 5182), and then displayed and recorded with a 2.5-GHz oscilloscope (LeCroy, WaveRunner 625Zi; Teledyne). An LED lamp (Thorlabs; M530L4, 530 nm) having a luminous flux density of 1.00 mW $cm^{-2}$ was used for stability and charge/discharge measurements. The power density was recorded using a spectrometer (Optimum, SRI-2000); the power density was corrected by using a Newport UV 818-L instrument.

**Table S1.** Performance of solution-processed photodiodes prepared in recent years.

| Donor: Acceptor | Dark current (A cm$^{-2}$) (bias) | R (A W$^{-1}$) (bias) | D* (Jones) | LDR (dB) (bias) | Cut-off ($f_{-3dB}$) (kHz) | Reference |
|---|---|---|---|---|---|---|
| PM6: Y6/ PC$_{71}$BM:P3HT | 3×10$^{-8}$ (-5V) | - | 6.8×10$^{12}$ (-5V) | 158 (-5V) | - | S1 |
| PTQ10: O-FBR | 1.7×10$^{-10}$ (-2V) | 0.34 (-2V) | 9.6×10$^{12}$ (-2V) | 72 (-2V) | 110 (-5V) | S2 |
| PBDTTT-EFT: eh-IDTBR | 1.13×10$^{-9}$ (-2V) | - | 1.63×10$^{13}$ (-1V) | 143 (-1V) | - | S3 |
| P3HT: IDTBR | 3.4×10$^{-8}$ (-5V) | 0.42 (-5V) | 3.98×10$^{12}$ (-5V) | - | 52.6 (-5V) | S4 |
| PBDTT-8ttTPD: PC$_{71}$BM | 8×10$^{-9}$ (-2V) | 0.38 (-2V) | 1×10$^{13}$ (-2V) | - | - | S5 |
| P3HT: indigo derivative | 9×10$^{-9}$/ 2.9×10$^{-8}$ (-1/-3V) | 0.4 (-3V) | ~1×10$^{12}$ (-1V) | ~170 (0/-1/-3V) | ~38 (0/-1/-3V) | S6 |
| PTB7-Th: COTIC-4F | 1.4×10$^{-8}$/ 1.6×10$^{-5}$ (0/-3V) | 0.37/ 0.42 (0/-3V) | 1.7×10$^{11}$/ 5.9×10$^{9}$ (0/-3V) | - | - | S7 |
| PTB7-Th: CO1-4F | 8.9×10$^{-10}$/ 8.0×10$^{-5}$ (0/-3V) | 0.46/ 0.52 (0/-3V) | 1.5×10$^{12}$/ 2.6×10$^{10}$ (0/-3V) | - | - | S7 |
| PTB7-Th: CTIC-4F | 1.3×10$^{-8}$/ 5.4×10$^{-6}$ (0/-3V) | 0.49/ 0.51 (0/-3V) | 7×10$^{11}$/ 1.2×10$^{10}$ (0/-3V) | - | - | S7 |
| PTB7-Th: CO$_{i8}$DFIC: PC$_{71}$BM | - | 0.35 (0V) | 5.6×10$^{11}$ (0V) | 135 (0V) | 2000 (0V) | S8 |
| PTB7-Th: CO1-4Cl | 5.8×10$^{-10}$/ 7×10$^{-9}$ (-0.1/-2V) | 0.45/ 0.52 (-0.1/-2V) | 3.31×10$^{13}$/ >1×10$^{13}$ (-0.1/-2V) | 126 (-0.1V) | 240 (-2V) | S9 |
| PTB7-Th: IFIC-i-4F: PC$_{71}$BM | 1.23×10$^{-11}$ (0V) | 0.38 (0V) | 1.93×10$^{14}$ (0V) | 80 (0V) | - | S10 |
| BDT-Th-3T: ITIC | ~1×10$^{-9}$ (-0.5V) | 0.31 (-0.5V) | 1.4×10$^{13}$ (-0.5V) | 232 (-0.5V) | 12 (-0.5V) | S11 |
| PFT-OEHp: Y6 | 5.8×10$^{-9}$/ 1.0×10$^{-7}$ (-0.1/-1 V) | 0.5/ 0.5 (-0.1/-1V) | >1×10$^{13}$/ 2.7×10$^{12}$ (-0.1/-1 V) | 135 (-0.1V) | >50 (-0.1V) | S12 |
| P3HT: ICBA | 1.2×10$^{-12}$ (0V) | 0.268 (0V) | 1.8×10$^{12}$ (0V) | >160 (0V) | 15 (0V) | S13 |
| PTNT: PC$_{71}$BM | 10$^{-5}$ (-0.5V) | 0.24 (0V) | 1.4×10$^{9}$ (0V) | 60 (0V) | 760 (0V) | S14 |
| P3HT: TV-ITIC | ~2×10$^{-2}$ (-20V) | - | 2.3×10$^{12}$ (-20V) | 151 (-20V) | 0.14 (-20V) | S15 |

| | | | | | | |
|---|---|---|---|---|---|---|
| P3HT: ETBI-F | ~$10^{-5}$ (-20V) | | $9.5\times10^{12}$ (-20V) | 100 (-20V) | | S16 |
| P3HT: $PC_{61}BM$ (with blocking layer) | $9\times10^{-5}$ (-2V) | 0.214 (-2V) | $6.59\times10^{10}$ (-2V) | >60 (-2V) | 864 (-2V) | S17 |
| PTB7-Th: IEICO-4F | $5.1\times10^{-10}$ (-2V) | 0.41 (-2V) | $5.6\times10^{13}$ (-2V) | 133 (-2V) | - | S18 |
| PNIR: ITIC | $6.1\times10^{-8}$ (-0.5V) | 0.1 (-0.5V) | $3.31\times10^{11}$ (-0.5V) | 58 (-0.5V) | - | S19 |
| PTB7-Th: CO1-4Cl | $1\times10^{-10}$ (-1V) | - | $5.82\times10^{12}$ (-1V) | - | 108.7 (-1V) | S20 |
| PM6:BTP-eC9: $PC_{71}BM$ | $3.27\times10^{-9}$ (-1V) | 0.59 (-1.5V) | $3.4\times10^{14}$ (0V) $1.1\times10^{13}$ (-1.5V) | 142 102 (-1V) | 450/610 (-1V) | This work |

**Table S2.** Contact angles and surface energies of the materials.

| | PM6 | BTP-eC9 | $PC_{71}BM$ |
|---|---|---|---|
| Surface Tensions ($\gamma$), mN $m^{-1}$ | 38.92 | 46.70 | 52.92 |
| Water Contact angle, deg. | 93.94 | 91.02 | 89.92 |
| DIM Contact angle, deg. | 50.46 | 33.40 | 12.34 |
| Interaction Parameters ($\chi$) | PM6: BTP-eC9 | | 0.35 |
| | PM6: $PC_{71}BM$ | | 1.07 |
| | $PC_{71}BM$:BTP-eC9 | | 0.19 |

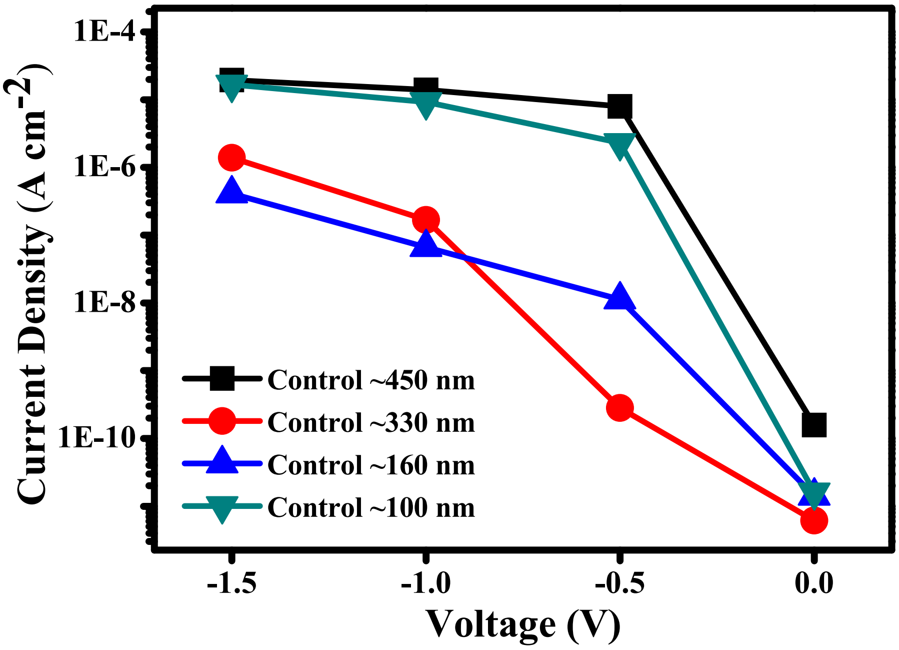


**Figure S1.** Dark currents of binary devices of various thicknesses.

**Table S3.** Blend layer thicknesses obtained at various deposition speeds.

| | Control | 0.1–PCBM | 0.5–PCBM | 1–PCBM |
|---|---|---|---|---|
| Spinning Speed (rpm) | Thickness (by α-step) | | | |
| 0.7 K | ~450 nm | ~420 nm | ~350 nm | ~400 nm |
| 1 K | ~330 nm | ~330 nm | ~330 nm | ~340 nm |
| 3 K | ~160 nm | ~150 nm | ~160 nm | ~140 nm |
| 5 K | ~100 nm | ~110 nm | ~100 nm | ~110 nm |

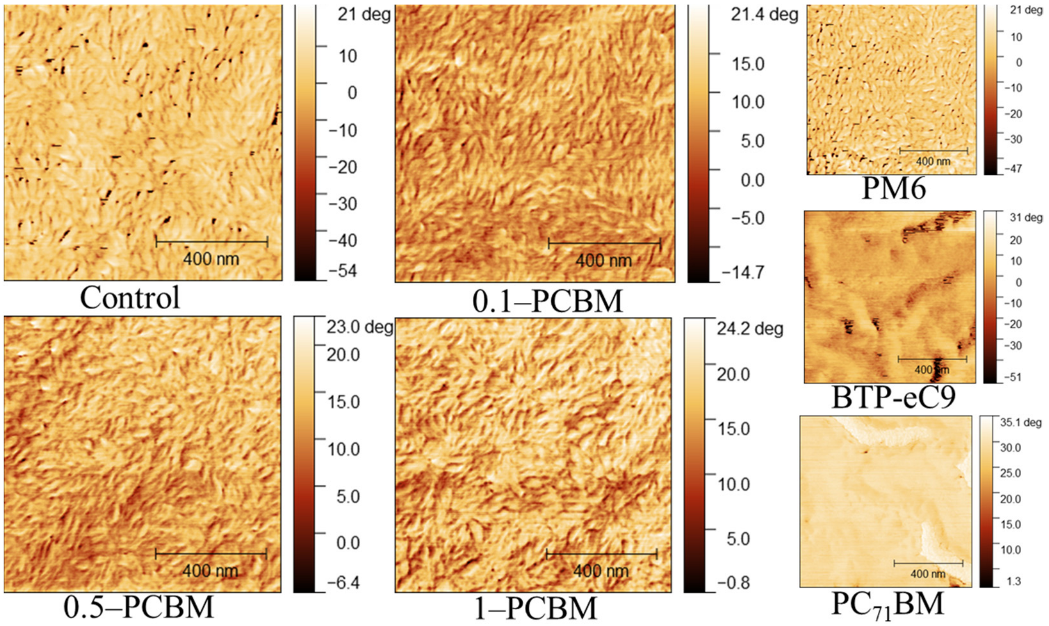


**Figure S2.** Tapping-mode AFM phase images of blend films prepared with various ratios of $PC_{71}BM$.

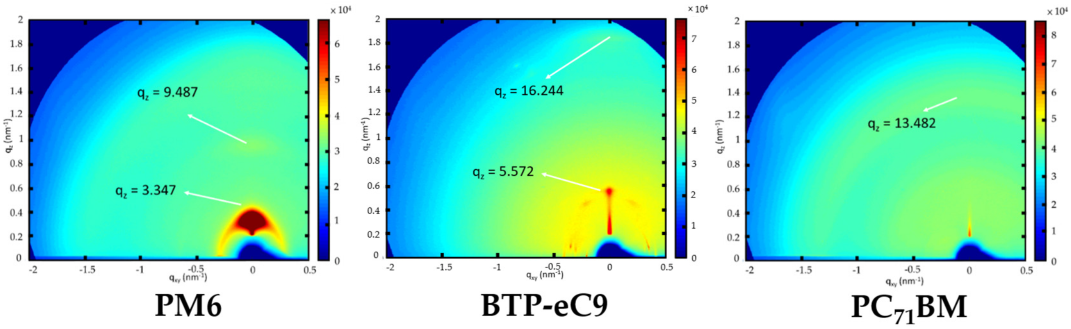


**Figure S3.** GIWAXS image of thin films of the pure materials.

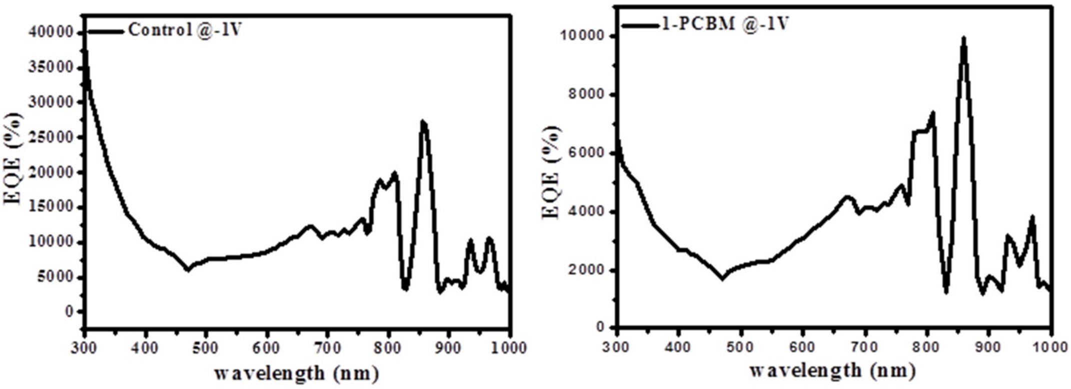


**Figure S4.** EQE spectra of OPDs.

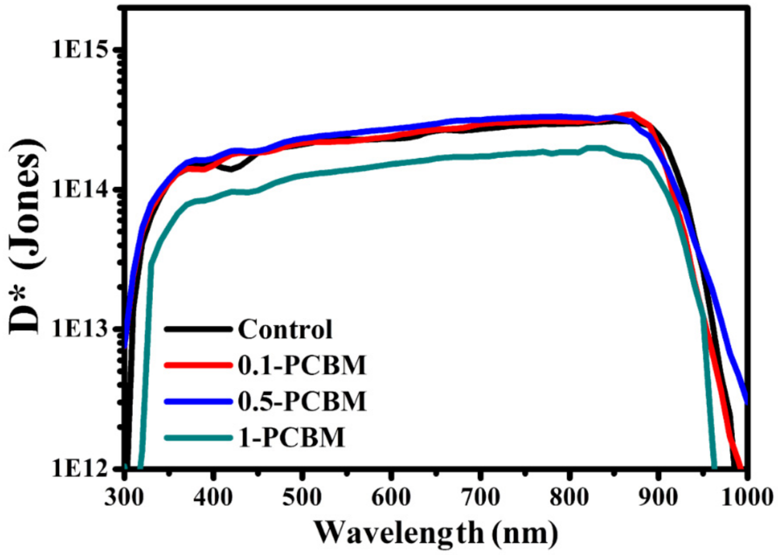


**Figure S5**. Plots of *D** for the OPDs at zero bias.

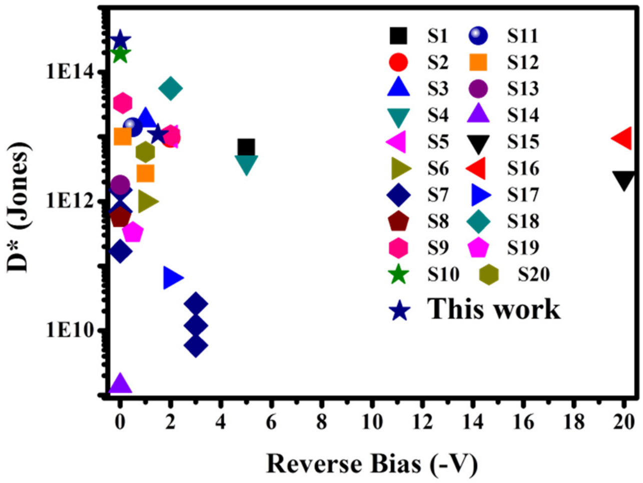


**Figure S6**. Reported detectivities of solution-processed organic photodiodes.

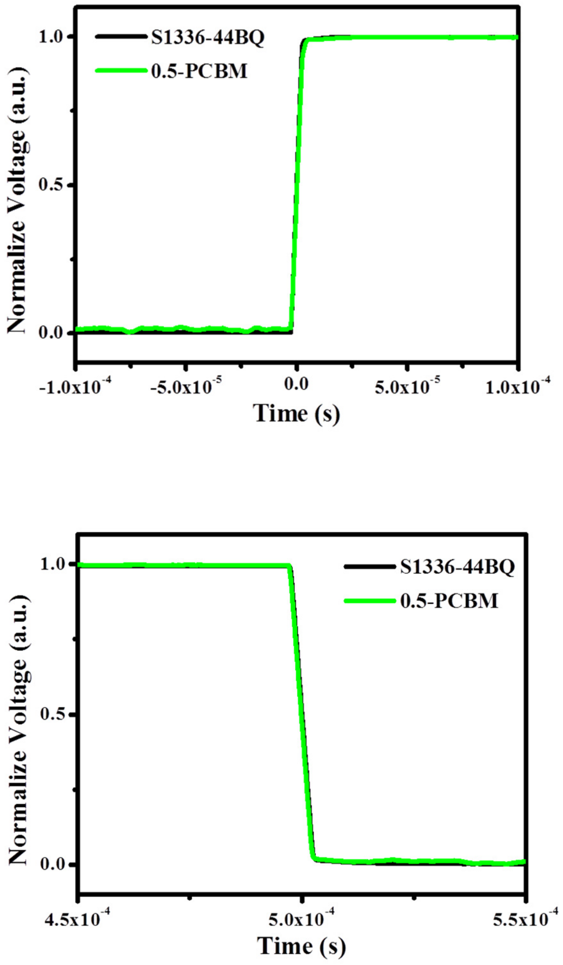


**Figure S**7. Rise and fall times of the small-area device (0.04 cm$^2$).

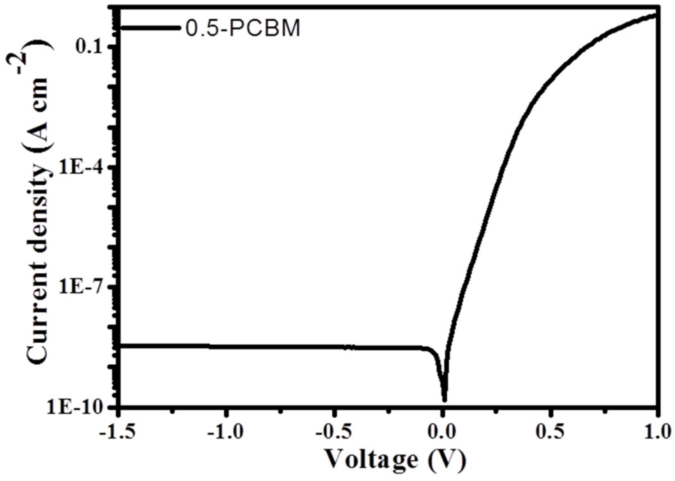


**Figure S**8. Dark current of the 0.5–PCBM device.

**Table S4.** Rise and fall times of Si-PD and 0.5–PCBM devices under a light source at 530 nm, 1 kHz, and a bias of −1 V.

.

| | Si (S1336–44BQ) | 0.5–PCBM |
|---|---|---|
| Rise time | 588.4 ns | 114 ns |
| Fall time | 594.6 ns | 100.3 ns |

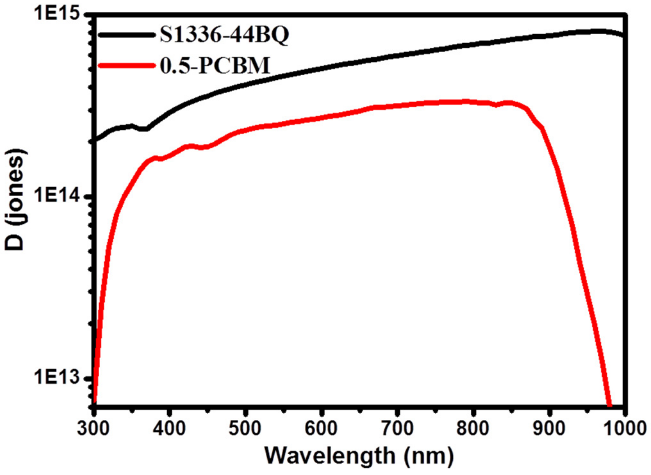


**Figure S9.** Plots of $D^*$ for the Si-PD and 0.5–PCBM devices.

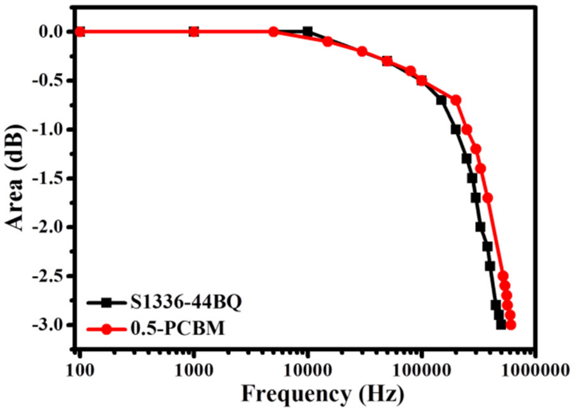


**Figure S10.** Cut-off frequencies of the Si-PD and 0.5–PCBM devices.
